# The STAT3 inhibitor GPB730 enhances the sensitivity to enzalutamide in prostate cancer cells

**DOI:** 10.1016/j.tranon.2022.101495

**Published:** 2022-07-30

**Authors:** Rebecka Hellsten, Anna Stiehm, Macarena Palominos, Margareta Persson, Anders Bjartell

**Affiliations:** a Department of Translational Medicine, Lund University, Scheelevägen 8, Building 404:A3, Lund SE-223 63, Sweden; b Department of Laboratory Medicine, Lund University, Scheelevägen 8, Building 404:A3, Lund SE-223 63, Sweden; c Department of Urology, Skåne University Hospital, Jan Waldenströms gata 5, Malmö SE-205 02, Sweden

**Keywords:** Prostate cancer, STAT3, Small molecule inhibitor, Enzalutamide, Combination therapy, CRPC, AR, androgen receptor, CI, combination index, CRPC, castration-resistant prostate cancer, IL-6, interleukin-6, PSA, prostate specific antigen, pSTAT3-S727, STAT3 phosphorylated at serine 727, pSTAT3-T705, STAT3 phosphorylated at tyrosine 705, STAT3, signal transducer and activator of transcription 3

## Abstract

Enzalutamide is a second-generation anti-androgen which has shown increased survival in patients with metastatic prostate cancer. However, some patients do not respond to this therapy or will develop resistance to treatment over time. Signal Transducer and Activator of Transcription 3 (STAT3) is known to be involved in castration-resistant prostate cancer and to interact with androgen receptor (AR)-signaling. This study aims to investigate the combination enzalutamide and the small molecule STAT3 inhibitor GPB730 for enhanced therapeutic effect in advanced prostate cancer *in vitro.* The prostate cancer cell lines LNCaP (androgen dependent) and C4–2 (androgen insensitive) were used. The effect of enzalutamide and GPB730, alone and in combination, was investigated on viability and IC_50_ values calculated. Enzalutamide and GPB730 treated LNCaP and C4–2 cells were subjected to western blot and QPCR analyses in order to investigate the expression of AR, STAT3 and down-stream targets. C4–2 were less sensitive to growth inhibition by enzalutamide than LNCaP cells. GPB730 enhanced the growth inhibitory effect of enzalutamide in LNCaP and C4–2 cells. The addition of GPB730 to enzalutamide decreased the IC_50_ values for enzalutamide by 3.3-fold for LNCaP and by 12-fold for C4–2. In C4–2 cells, GPB730 alone decreased PSA expression and enhanced the enzalutamide induced decrease in NKX3.1 expression. GPB730 and enzalutamide in combination enhanced inhibition of c-myc and survivin expression. This study suggests that enzalutamide may be combined with the STAT3 inhibitor GPB730 in order to enhance the efficacy of enzalutamide, offering a new therapeutic approach in advanced prostate cancer.

## Highlights

• STAT3 inhibitor GPB730 and enzalutamide in combination synergistically inhibited growth *in vitro*.
• GPB730 and enzalutamide in combination enhanced inhibition of c-myc and surviving expression.
• GPB730 showed an inhibitory effect on androgen receptor regulated genes.
• Enzalutamide combined with GPB730 may enhance the therapeutic effect of antiandrogen treatment.

## Introduction

Although the incidence of prostate cancer is higher compared to the mortality, there is an urgent need to decrease the death rate [1,2]. A recent but modest decrease in mortality rates can be explained by early detection and therapeutic advances, however there is still a need for novel treatments in men with castration-resistant prostate cancer (CRPC) and when chemotherapy and other therapies have failed. Enzalutamide is a second-generation anti-androgen which has shown significant therapeutic effect in prostate cancer patients at different stages of the disease. However, there is a group of patients that responds poorly and those who do will eventually develop resistance and progress [3,4]. Enzalutamide has been investigated in combination with established therapeutics such as PARP-inhibitors, glucocorticoid receptor antagonists, radium-223 and immune checkpoint inhibitors with varying efficacy and outcome [5,6,7,8]. Thus there is a need for additional approaches to enhance the therapeutic effect of enzalutamide.

The mechanisms behind intrinsic and acquired resistance to enzalutamide include activation of signaling pathways such as MAPK and PI3K/AKT, cytokine dysregulation in particular IL-6 signaling via JAK/STAT3, the expression of androgen receptor (AR) splice variants such as ARV7, but also other mechanisms are possible [9,10,11,12,13].

The transcription factor signal transducer and activator of transcription 3 (STAT3) has been associated with aggressive tumor growth, reduced androgen sensitivity and metastatic spread in prostate cancer [14]. High levels of active (phosphorylated) STAT3 (pSTAT3) have been detected in metastases of CRPC patients [15]. The IL-6/JAK/STAT3 pathway has been shown to be enriched in metastatic CRPC patients with poor response to enzalutamide treatment [16]. In addition, increased levels of STAT3 have been observed in experimental models of CRPC and related to anti-androgen resistance and loss of AR [17,18,19,20]. Activation of STAT3 has been implicated in resistance to anti-androgens such as enzalutamide and bicalutamide, and inhibition of STAT3 may reverse this resistance [18]. Thus targeting STAT3 in combination with enzalutamide has the potential of enhancing therapeutic efficacy and improving clinical outcome. However, there are no STAT3 inhibitors in clinical use as of today, thus there is a need for exploratory studies in a preclinical setting.

We have previously demonstrated a growth inhibitory effect of the small molecule STAT3 inhibitor galiellalactone as monotherapy in various prostate cancer models where STAT3 is constitutively activated in the tumor cells [21,22,23,24]. In addition, we have shown that a synthetic analogue of galiellalactone, GPB730 [25], improved the therapeutic efficacy of immunotherapy in a prostate cancer *in vivo* model [26].

The aim of this study was to investigate if a combination of enzalutamide and the small molecule STAT3 inhibitor GPB730 can enhance the therapeutic effect of enzalutamide in advanced prostate cancer.

## Materials and methods

### Cell lines and reagents

The human prostate cancer cell lines LNCaP, C4–2 and DU145 (from the American Type culture Collection [ATCC]) were used. LNCaP and DU145 cells were cultured in RPMI-1640 medium supplemented with 10% FBS, 100 units/ml penicillin and 100 mg/ml streptomycin. C42 cells were cultured in RPMI-1640 medium supplemented with 10% FBS, 1% glutamax, 1% sodium pyruvate, 1% non-essential amino acids, 100 units/ml penicillin and 100 mg/ml streptomycin. All cells were incubated at 37 °C in a humidified atmosphere of 95% O_2_ and 5% CO_2_. C4–2 is an androgen independent prostate cancer cell line derived from of the parental androgen dependent cell line LNCaP [27]. Cells were routinely tested for the abcence of mycoplasma (Eurofins genomics). Cell lines were authenticated by short tandem repeat profiling (ATCC).

The STAT3 inhibitor GPB730 was provided by Glactone Pharma AB (Gothenburg, Sweden). The compound was stored at −20 °C diluted in DMSO to 0.05 M. A working solution of 1 mM was prepared by dilution in PBS. Enzalutamide (MedChemExpress) was diluted to 0.05 M in DMSO and stored at −80 °C.

### Western blot analysis

LNCaP and C4–2 cells were treated with GPB730 and enzalutamide for 72 h. Cell lysates were prepared and western blot analysis was performed according to previous publication [21,22]. Primary antibodies used were anti-STAT3 (#4904 Cell Signaling Technology), anti-pSTAT3-T705 (ab76315 Abcam), anti-pSTAT3-S727 (#9134 Cell Signaling Technology), c-myc (ab32072 Abcam), survivin (#2808 Cell Signaling Technology), PSA (sc7638 Santa Cruz) and androgen receptor (ab108341 Abcam). Beta-actin (#A5441 Sigma) was used as loading control. LNCaP cells stimulated with 50 ng/ml IL-6 for 30 min and DU145 cells were used as positive controls for pSTAT3-S727 and pSTAT3-T705 expression.

### Viability and apoptosis assay and calculation of drug interactions

The proliferation and viability of LNCaP and C4–2 cells treated with GPB730 and enzalutamide was measured using CellTiter Glo (Promega). Cells were seeded in 96-well plates (opaque clear bottom, Corning) at a density of 5 000 cells/well. Cells were treated with GPB730 or enzalutamide at concentrations 1 μM, 3 μM, 5 μM, 10 μM and 30 μM for 72 h. For combination studies the concentrations used are indicated in the text for the specific experiments. Drug interactions were determined by calculating the combination index (CI) by CompuSyn software (CombuSyn, Inc.) using the Chou-Talalay method [28] at non-constant ratios.

Apoptosis as measured by caspase 3/7 activity using the ApoTox Triplex assay (Promega). C4–2 and LNCaP cells were cultured in 96-well plates (opaque clear bottom) and treated with GPB730 and enzalutamide for 48 h. The assay was performed according to the manufacturer's protocol. The results of the viability and apoptosis assays are presented as percent of untreated control cells.

### Quantitative real-time PCR

The expression of genes related to the androgen receptor and STAT3 were investigated in prostate cancer cell lines with quantitative real-time PCR (QPCR). The mRNA expression was investigated after 48 h of treatment with GPB30 and enzalutamide as single or combination treatment. The RNA was extracted with the use of a RNAeasy kit (Qiagen). For internal control the housekeeping genes YWHAZ and GAPDH were used. Gene expression was normalized against the mean value of the housekeeping genes to determine the relative expression levels of the genes of interest. Genes that were analyzed were androgen receptor full-length (AR-FL), prostate specific antigen (PSA), NKX3.1, STAT3, survivin and c-myc. The primer sequences are presented in Table S1.

### Statistical analyses

Statistical analysis was performed using GraphPad Prism. Differences between treatment groups was analyzed by one-way ANOVA followed by Tukey's multiple comparisons tests. IC_50_ values were calculated using non-linear regression analysis in GraphPad prism. Data are presented as ± standard error of the mean (SEM). Statistical significance was considered when *p* ≤ 0.05.

## Results

### GPB730 and enzalutamide synergistically inhibit prostate cancer cell viability and growth

**Fig. 1:**
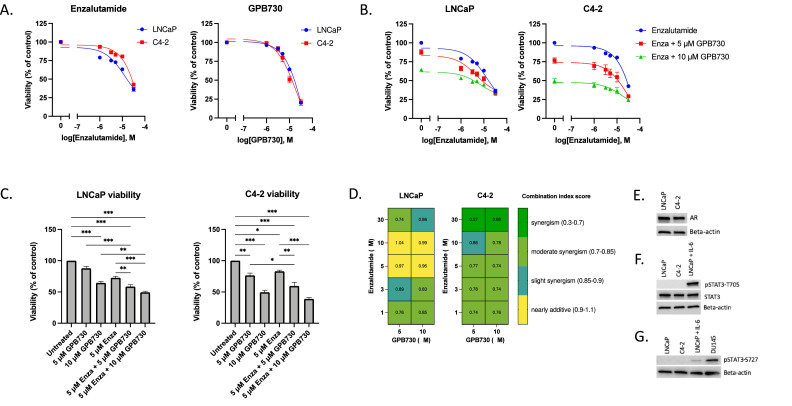
*Effect of enzalutamide and GPB730 on the viability of the prostate cancer cell lines LNCaP and C4–2 A*. Dose-response curves for enzalutamide and GPB730 in LNCaP and C4–2 cells treated for 72 h (*n* = 3–4). **B.** Dose-response curves for enzalutamide with and without the addition of 5 μM or 10 μM GPB730 in LNCaP and C4–2 cells treated for 72 h (*n* = 3–4). **C.** Viability of LNCaP and C4–2 cell treated with enzalutamide and GPB730, alone or in combination (*n* = 3–4). The viability was calculated as percent of untreated control and presented as mean ± SEM. * *p* ≤ 0.05; ** *p* ≤ 0.01; *** *p* ≤ 0.001. **D**. Combination index (CI) values for enzalutamide and GPB730 in LNCaP and C4–2 cells treated for 72 h. **E–G.** Western blot analysis of protein expression levels of AR, pSTAT3-S727, pSTAT3-T705 and STAT3 in LNCaP and C4–2 cells. IL-6 stimulated LNCaP cells and DU145 cells were used as positive controls for pSTAT3-T705 and pSTAT3-S727 expression.

The viability of the prostate cancer cell lines LNCaP and C4–2 treated with enzalutamide or GPB730 was investigated and the IC_50_ values calculated (Table 1, Fig. 1A). C4–2 cells were less sensitive to growth inhibition by enzalutamide than LNCaP cells with an IC_50_ nearly twice as high (27 μM and 14 μM, respectively). LNCaP and C4–2 cells showed similar sensitivity to growth inhibition by the STAT3 inhibitor GPB730 (IC_50_ 16 μM and 13 μM, respectively). LNCaP and C4–2 cells both express AR and STAT3 at similar levels but do not constitutively express pSTAT3-T705 or pSTAT3-S727 (Fig. 1E, F).

**Table 1:** *IC_50_ values for enzalutamide and GPB730 in LNCaP and C4–2 cells*. The IC_50_ values for enzalutamide and GPB730 were calculated based on the viability of cells treated for 72 h.

	LNCaP	C4–2
**IC_50_ Enzalutamide**	14.3 μM	27.1 μM
**IC_50_ GPB730**	16.5 μM	13.4 μM
**IC_50_ Enzalutamide + 5 μM GPB730**	7.7 μM	8.1 μM
**IC_50_ Enzalutamide + 10 μM GPB730**	4.2 μM	2.3 μM

Next, we investigated whether GPB730 would enhance the growth inhibitory effect of enzalutamide. When adding GPB730 to enzalutamide the growth inhibition was significantly enhanced in both LNCaP and C4–2 cells compared to enzalutamide or GPB730 alone (Fig. 1B, C). In the presence of 10 μM GPB730 the IC_50_ value of enzalutamide was decreased by 12-fold in C4–2 cells (from 27.1 uM to 2.3 μM) and by 3.3-fold in LNCaP cells (from 14.3 μM to 4.2 μM) (Table 1). Calculation of the combination index (CI) reveal a synergistic growth inhibitory effect of enzalutamide and GPB730 in C4–2 cells at all dose combinations investigated (Fig. 1D). A synergistic or additive growth inhibitory effect of enzalutamide and GPB730 was observed in LNCaP cells depending on dose.

### GPB730 induces apoptosis in prostate cancer cells

**Fig. 2:**
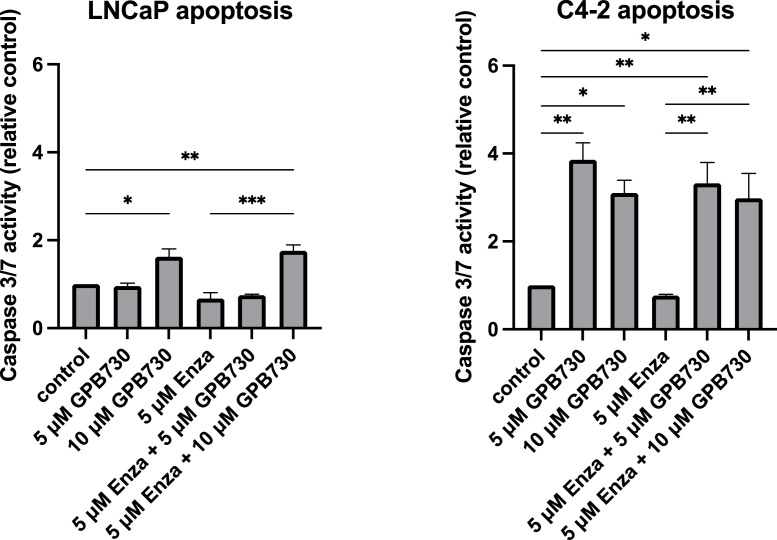
*GPB730 induces apotosis in prostate cancer cells***.** LNCaP and C-42 cells were treated with GPB730 and enzalutamide for 48 h. Apotosis was measured as Caspase 3/7 activity relative untreated control and presented as mean ± SEM. * *p* ≤ 0.05; ** *p* ≤ 0.01; *** *p* ≤ 0.001. *N* = 3.

Apoptosis was measured as caspase 3/7 activity in LNCaP and C4–2 cells treated with enzalutamide and GPB730 for 48 h. Enzalutamide treatment alone did not induce apoptosis in LNCaP or C4–2 cells (Fig. 2). However, GPB730 significantly increased apoptosis in both cell lines, with the most prominent effect in C4–2 cells where an increase in apoptosis was observed even at the lower concentrations of GPB730. No additional increase in apoptosis was observed with the combination enzalutamide and GB730.

### Effect of GPB730 and enzalutamide on androgen receptor expression and activity

**Fig. 3:**
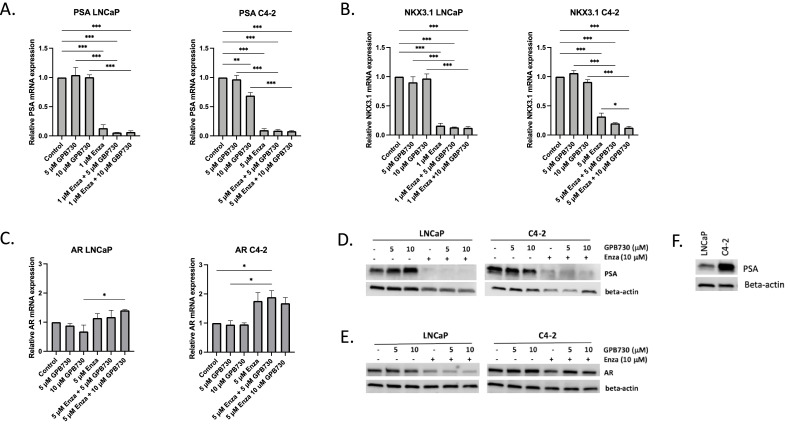
*The effect of GPB730 and enzalutamide on AR expression and activity in LNCaP and C4–2 cells***. A–C** mRNA expression of AR and the AR regulated genes PSA and NKX3.1 in LNCaP and C4–2 cells treated with enzalutamide and GPB730 for 48 h. The mRNA expression levels were calculated as percent of untreated control and presented as mean ± SEM. * *p* ≤ 0.05; ** *p* ≤ 0.01; *** *p* ≤ 0.001. *N* = 3. D, **E**. Western blot analysis of AR and PSA protein expression in LNCaP and C4–2 cells treated with enzalutamide and GPB730 for 72 h. **F.** PSA protein levels in LNCaP and C4–2 cells.

Next, we investigated the effect of GPB730 and enzalutamide on AR expression and activity measured as expression of the AR regulated genes PSA and NKX3.1 (Fig. 3). As expected, enzalutamide inhibited AR activity in both LNCaP and C4–2 cells as observed by a drastic decrease in PSA mRNA and protein expression (Fig. 3A, D) and decrease in NKX3.1 mRNA expression (Fig. 3B). GPB730 alone decreased PSA mRNA and protein expression in C4–2 cells which was not observed in LNCaP cells (Fig. 3A, D). C4–2 cells showed higher expression of PSA protein than LNCaP cells (Fig. 3F). Addition of GPB730 to enzalutamide did not affect the already low levels of PSA mRNA expression in either cell line although an additional decrease was observed at the protein level (Fig. 3A, D). GPB730 alone did not affect NKX3.1 expression in either cell line (Fig. 3B). However, in C4–2 cells the combination GPB730 and enzalutamide significantly reduced the expression of NKX3.1 compared to enzalutamide alone.

The AR protein expression was unaffected by GPB730 and a slight decrease was observed by enzalutamide treatment in both LNCaP and C4–2 cells (Fig. 3E). AR mRNA expression levels were not affected by enzalutamide or GPB730 alone (Fig. 3C). However, AR mRNA levels were significantly increased by enzalutamide in combination with GPB730 in both cell lines which was not reflected at the AR protein levels (Fig. 3C, E).

### Effect of GPB730 and enzalutamide on the expression of STAT3 and STAT3 regulated genes

**Fig. 4:**
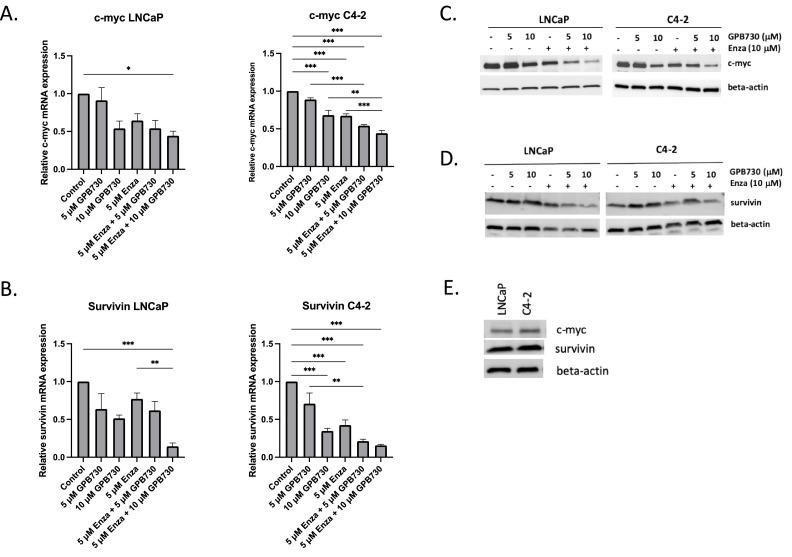
*Effect of GPB730 and enzalutamide on STAT3 regulated genes***. A, B.** mRNA expression of the STAT3 regulated genes c-myc and survivin in LNCaP and C4–2 cells treated with enzalutamide and GPB730 for 48 h. The mRNA expression levels were calculated as percent of untreated control and presented as mean ± SEM. * *p* ≤ 0.05; ** *p* ≤ 0.01; ***p ≤ 0.001. *N* = 3. **C, D.** Western blot analysis of c-myc and survivin protein expression in LNCaP and C4–2 cells treated with enzalutamide and GPB730 for 72 h. **E.** C-myc and PSA protein levels in LNCaP and C4–2 cells.

**Fig. 5:**
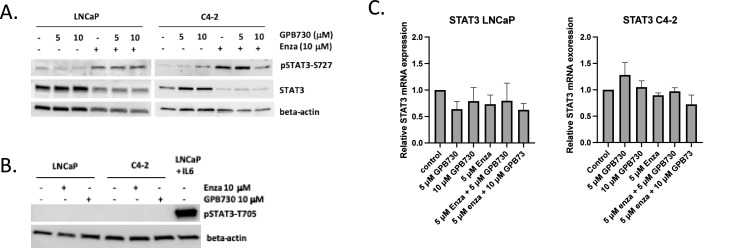
*Effect of GPB730 and enzalutamide on STAT3 expression and phosphorylation status*. **A,B.** Western blot analysis of STAT3, pSTAT3-S727 and pSTAT3-T705 in LNCaP and C4–2 cells treated with enzalutamide and GPB730 for 72 h. IL-6 stimulated LNCaP cells were used as positive control for pSTAT3-T705 expression. **C.** mRNA expression analysis of the STAT3 regulated genes c-myc and survivin in LNCaP and C4–2 cell treated with enzalutamide and GPB730 for 48 h. The mRNA expression levels were calculated as percent of untreated control and presented mean ± SEM. *N* = 3.

Next we investigated the effects of enzalutamide and GPB730 on the expression of STAT3 protein and the phosphorylated forms of STAT3 (pSTAT-S727 and pSTAT3-T705) and on STAT3 regulated genes (Figs. 4 and 5).

C-myc gene and protein expression was decreased by both enzalutamide and GPB730 alone in C4–2 cells (Fig. 4A, C). Enzalutamide in combination with GPB730 provided an additional decrease in c-myc gene expression in C4–2 cells compared to either compound alone (Fig. 4A). The decrease in c-myc expression was confirmed at the protein level (Fig. 4C). A similar effect on c-myc protein expression was observed in LNCaP cells where the combination enzalutamide and GPB730 provided a pronounced decrease in c-myc protein expression compared to untreated control and each compound alone (Fig. 4C). C-myc mRNA expression in LNCaP cells was only significantly decreased by the combination enzalutamide and GPB730 (Fig. 4A).

The expression of the STAT3 regulated gene survivin was significantly reduced in C4–2 cells by GPB730 and enzalutamide, each alone and in combination (Fig. 4B). In LNCaP cells, survivin mRNA expression was only significantly decreased by the combination enzalutamide and GPB730 (Fig. 4B). The combination enzalutamide and GPB730 reduced survivin protein expression in both cell lines (Fig. 4D).

LNCaP and C4–2 cells express equal protein levels of survivin. C-myc protein is slightly higher expressed in C4–2 than in LNCaP cells (Fig. 4E).

Enzalutamide induced pSTAT3-S727 expression in both LNCaP and C4–2 cells (Fig. 5A). pSTAT3-T705 was not constitutively expressed or induced by enzalutamide in either cell line (Fig. 5B). Enzalutamide decreased the total amount of STAT3 protein although no change in mRNA expression was observed (Fig. 5A, C). The STAT3 protein expression and the phosphorylation of STAT3 was not affected by GPB730 treatment in LNCaP or C4–2 cells (Fig. 5A, B).

## Discussion

There is an unmet need for treatment of patients with CRPC. The results of this study suggest that combination therapy with the anti-androgen enzalutamide and the STAT3 inhibitor GPB730 may be a promising therapeutic alternative. In this study we show that enzalutamide and GPB730 in combination provide synergistic growth inhibitory effects in an androgen independent cell line and synergistic or additive growth inhibitory effect in an androgen dependent cell line *in vitro*. In addition, the combination further decreased the expression of c-myc which is an interesting therapeutic target in prostate cancer. In the androgen insensitive cell line C4–2, GPB730 showed an inhibitory effect on AR regulated genes. The addition of GPB730 to enzalutamide may thus enhance the efficacy of anti-androgen therapy.

In this study we used the androgen sensitive prostate cancer cell line LNCaP and the androgen insensitive cell line C4–2. The C4–2 cells are derived from LNCaP xenografts grown in castrated mice providing a model of CRPC [27]. C4–2 cells have ligand independent AR activation and AR is constitutively activated even in the absence of androgens [29]. C4–2 cells are less sensitive to enzalutamide than the parental LNCaP cells in terms of viability [30,31], which is in accordance with our observations. The N-terminal domain (NTD) is shown to be important for the androgen independent AR activity in C4–2 cells [29]. Interestingly, this region is also shown to interact with STAT3 [32]. In LNCaP cells STAT3 interaction with the NTD of AR leads to androgen independent activation in the presence of IL-6 [32].

STAT3 interaction with AR may lead to increased AR protein stability and enhanced transcriptional activity, promoted by serine phosphorylation of STAT3 [33]. Phosphorylation of STAT3 at serine 727 mediates AR-STAT3 interaction thus potentially preventing full inactivation of AR by enzalutamide. Inhibition of AR-STAT3 interaction may thus enhance the inhibitory effect of enzalutamide on AR activity. Indeed, a further decrease in the AR regulated NKX3.1 was observed in C4–2 cells by the combination GPB730 and ezalutamide. In addition, the decrease in PSA by GPB730 may reflect STAT3 as a co-factor for AR activity in C4–2 cells. Considering that C4–2 cells are dependent on the NTD for androgen independent AR activation and that his region may interact with STAT3, this may be reflected in the observation that C4–2 cells showed a greater response to STAT3 inhibition and enhanced inhibitory effect when combined with enzalutamide.

In addition to resistance mechanisms involving activated signaling pathways and expression of AR splice variants, the sensitivity to anti-androgens is also associated with AR protein stability [34]. The AR is observed to be more stable in C4–2 cells than in LNCaP cells, suggesting a mechanism for the cells lines different sensitivities to enzalutamide.

Enzalutamide induced the expression of pSTAT3-S727 in both LNCaP and C4–2 cells which is in line with a previous study on LNCaP cells [35]. Other studies have shown that phosphorylation at tyrosine 705 is associated with AR loss, anti-androgen resistance, androgen deprivation or ADT [18,19,36] although we did not observe a constitutive or induced expression of pSTAT3-T705 in the cell lines used in the current study. Interestingly, enzalutamide reduced the expression of STAT3 protein in both cell lines and there was no effect on the gene transcription of STAT3 itself.

GPB730 is a direct STAT3 inhibitor similar to galiellalactone which prevents STAT3 from binding to STAT3-DNA binding sequences and blocking transcription without significantly effecting phosphorylation [21,22,23]. The enzalutamide induced phosphorylation of STAT3 in LNCaP and C4–2 cells was not affected by GPB730 which is as expected. Instead, the STAT3 inhibitory effect of GPB730 was observed by inhibition of the STAT3 down-stream target genes c-myc and survivin. In addition, STAT3 can be transcriptionally active independent of phosphorylation status and unphosphorylated STAT3 (uSTAT3) may drive oncogenesis [37]. However, the role for uSTAT3 in CRPC needs to be clarified.

C4–2 cells were more responsive to STAT3 inhibition by GPB730 than LNCaP cells in terms of the inhibitory effect on STAT3 and AR related genes and the enhanced apoptotic activity. However, the overall combination effect with enzalutamide and GPB730 was similar in both cell lines.

AR may in turn affect the transcriptional activity of STAT3 and anti-androgens are observed to decrease the transcriptional activity of STAT3 in LNCaP cells [38]. This is in line with our observation that enzalutamide alone decreased the expression of STAT3 regulated genes. Enzalutamide in combination with GPB730 provided an additional inhibitory effect on STAT3 regulated genes.

C-myc is overexpressed in prostate cancer and is involved in disease progression and identified as an interesting therapeutic target [39,40]. C-myc and AR are shown to be positively correlated in CRPC samples [41]. In addition, c-myc is thought to be involved in resistance to enzalutamide and targeting c-myc sensitizes prostate cancer cells to enzalutamide [41]. C-myc is shown to be upregulated in enzalutamide-resistant LNCaP cells and targeting c-myc lead to increased cell death [42]. The c-myc gene expression is regulated by STAT3 [43]. In this study we observed a decrease in c-myc mRNA and protein expression in LNCaP and C4–2 cells by the STAT3 inhibitor GPB730 and the decrease was even further accentuated by the combination of enzalutamide and GPB730. c-Myc binds to regulatory regions of the AR gene to induce AR-gene transcription [41] and a decrease in c-myc may thus partly inhibit AR transcriptional activity, providing a mechanism for the observed enhanced effects. In addition, AR in turn may induce c-myc expression [41,44] possibly explaining the observed inhibitory effect of enzalutamide on c-myc expression. Targeting c-myc by GPB730 may thus provide a therapeutic approach in CRPC and may be a mechanism to enhance the efficacy of enzalutamide. C-myc has long been considered “undruggable” and attemps have been made using different approaches such as targeting cofactors, protein degradation and dimerization with varying results [45]. No direct c-myc inhibitor has yet been put into clinical use.

Another interesting target is survivin (BIRC5 gene), an anti-apoptotic protein which is transcriptionally regulated by STAT3. Survivin is identified as a target for cancer therapy as it is implicated in tumorigenesis [46]. In addition, survivin is shown to be involved in resistance to ADT in prostate cancer [47]. Survivin was downregulated by GPB730 with an even greater decrease when combined with enzaluatamide. This further strengthens the validity of combining GPB730 with enzalutamide in CRPC.

The observed synergistic effect of the combination enzalutamide and GPB730 in terms of decreased viability is likely due to the combined effect of targeting both AR activity and STAT3 gene expression in addition to inhibiting STAT3 as a possible cofactor for AR activity [33,35,32].

As the STAT3 pathway is observed to be enriched in non-responders to enzalutamide treatment in metastatic CRPC patients [16], patient selection and a well-founded decision on when to apply a therapy is crucial for a beneficial therapeutic outcome. A selection of patients with enhanced STAT3 activity at treatment start may be eligible to combination therapy with enzalutamide and STAT3 inhibitor in order to prevent or postpone resistance and to enhance treatment efficacy. This study showed an enhanced efficacy of enzalutamide when combined with the STAT3 inhibitor GPB730 in prostate cancer cells which have not previously been exposed to enzalutamide and focuses on the intrinsic resistance to enzalumide treatment. This offers an opportunity for selection of subgroups eligible for combining enzalutamide with STAT3 inhibition at treatment start, and not only when acquired resistance evolves.

A limitation to the study is that we do not take into consideration the whole tumor which includes the tumor microenvironment comprising of immune cells and secreted cytokines which play a role in therapy resistance. STAT3 is a central transcription factor involved in the cross-talk between tumor cells and immune cells in the tumor microenvironment [20]. Another limitation is that we only investigated a few cell lines and do not include long-term enzalutamide treated prostate cancer cells which have developed resistance over time. Furthermore, the underlying mechanisms of the observed enhanced effects needs further exploration.

## Conclusion

Taken together, this study suggests that enzalutamide may be combined with the STAT3 inhibitor GPB730 in order to enhance the efficacy of anti-androgen treatment, offering an additional therapeutic approach for advanced prostate cancer.

## CRediT authorship contribution statement

**Rebecka Hellsten:** Conceptualization, Visualization, Formal analysis, Funding acquisition, Writing – original draft, Writing – review & editing. **Anna Stiehm:** Investigation, Writing – review & editing. **Macarena Palominos:** Investigation, Writing – review & editing. **Margareta Persson:** Investigation, Writing – review & editing. **Anders Bjartell:** Conceptualization, Funding acquisition, Writing – review & editing.

## Declaration of Competing Interest

Anders Bjartell is a board member and share-holder in Glactone Pharma AB, Gothenburg, Sweden which provided the compound GPB730 used in this study. Rebecka Hellsten is a share-holder in Glactone Pharma AB, Gothenburg, Sweden which provided the compound GPB730 used in this study. Anna Stiehm, Macarena Palominos and Margareta Persson have no competing interests.

## Appendix Supplementary materials

Image, application 1
