# Supplementary material for: The STAT3 inhibitor GPB730 enhances the sensitivity to enzalutamide in prostate cancer cells
Source: Transl Oncol. 2022 Jul 30;24:101495. doi: 10.1016/j.tranon.2022.101495 (PMC9344336; doi:10.1016/j.tranon.2022.101495)
Supplement: Supplementary file 1 [file mmc1.docx]

**Table S1.** Primer sequences

| **Gene name** | **Primer sequences** |
| --- | --- |
| AR-FL | FW: ACATCCTGCTCAAGACGCTTCTACC  RV: CACTTGCACAGAGATGATCTCTGCC |
| PSA | FW: CAAGACTCAAGCCTCCCCAG  RV: GCCAGTATTCCCCAGGACAC |
| NKX3.1 | FW: AGTCCACTGAGCAAGCAA GG RV: TCTCCCCTCTACCAGCTCAC |
| c-myc | FW: CGTCTCCACACATCAGCACAA RV: CACTGTCCAACTTGACCCTCTTG |
| survivin | FW: ACCGCATCTCTACATTCAAG  RV: CAAGTCTGGCTCGTTCTC |
| STAT3 | FW: GCTTCCTGCAAGAGTCGAATG  RV: TGTAGAAGGCGTGATTCTTCCC |
| GAPDH | FW: TGCACCACCAACTGCTTAGC  RV: GGCATGGACTGTGGTCATGAG |
| YWHAZ | FW: ACTTTTGGTACATTGTGGCTTCAA  RV: CCGCCAGGACAAACCAGTAT |
